# Supplementary material for: Patterns of Intron Gain and Loss in Fungi
Source: PLoS Biol. 2004 Nov 30;2(12):e422. doi: 10.1371/journal.pbio.0020422 (PMC532390; doi:10.1371/journal.pbio.0020422)
Supplement: Table S1 — Also available at http://genes.mit.edu/NielsenEtAl/. (4.3 MB ZIP). [file pbio.0020422.st001.zip › NielsenEtAl/html/224.html]

AN6374.1.NCU07070.1.MG04993.1.FG02752.1


```
 CLUSTAL W (1.82) Multiple Sequence Alignments - Introns Inserted


Sequence 1: NCU07070.1	676 aa
Sequence 2: FG02752.1	615 aa
Sequence 3: MG04993.1	636 aa
Sequence 4: AN6374.1	610 aa
Alignment Length: 698 aa
Number Identitical Residues: 256 aa
Alignment Score (without introns) 13284


MG04993.1 	-MKRKLNENNEP-----------VLVSGTNKKKSDTEVSSA-------------------
NCU07070.1	MAKRKLNETDEPSVVTEPQTQKKTKKSSTEKKEKKEKKSKSQSVKPQEEKPEATQQQAQQ
FG02752.1 	------------------------MSASTKRKRDQAEESVP------AENPAST------
AN6374.1  	-MKRKLDANDVP---------------STEANEAEEVKGAD-------------------
          	  . . . .. .               .*: :. .   .                     

MG04993.1 	-------VANATPSSEAASSS--------------SFADLGLDPRLLQAVAQQSFQKPTL
NCU07070.1	QTEEEQELEQQLKDEQKQQDEKDEKKQSEKDDADLTFSDLGLDPRLVQAVAKQSFEKPTL
FG02752.1 	------DVEKAIKPAQKQEEE-------------TSFVDLGLDPRLLQAIAQQKFAKPTL
AN6374.1  	------------------RDE--------------DFETLNLDPRLRQALVKEKFSKPTP
          	                   ..               *  *.***** **:.::.* *** 

MG04993.1 	VQSKAIPLALEGRDVLA~KAKTGSGK0TAAYVLPILQAVLKRKQ0INPGATYISSLILVP
NCU07070.1	VQRKAIPLALAGQDVLC~KAKTGSGK~TAAYVLPVLSGILKRKA0TDP-TPFTSALILVP
FG02752.1 	VQRKAIPLALNGQDVLA~KADCGSGK~TAAYVLPLLSSILKRKA0TDS-TAFTTALILVP
AN6374.1  	VQAKAIPLALEGKDILA1RAKTGSGK~TAAYVLPILQTILQKKA0IDPSMKATTGLILVP
          	** ******* *:*:*. :*. **** *******:*. :*::*   :..    :.*****

MG04993.1 	TRELTVQVTKEVERFSAFCAKEVQVVGLTDKVSDAVQRSLLQSSSPDIVVSTPSTAWRNV
NCU07070.1	TRELADQVHKAIDAFSAFCTKDIQSAKLTDNVSDAVLRSLLANA-PDVIVSTPARAWHNI
FG02752.1 	TRELADQVSKAIEQFASFCAKDISTAKLTDKVSSKVQRALLSNS-PDIVISTPSTAWQNV
AN6374.1  	TRELAEQVQKVITTFAAFCGKDVRSVNLTQKVSDAVQKAMLADY-PDIVVSTPARVIANL
          	****: ** * :  *::** *::  . **::**. * :::* .  **:::***: .  *:

MG04993.1 	DSGALSLDKLTHLVLDEADLVLSYGYDEDLEKVARGLPKG-VQTVMTSATLT-DEIDTLK
NCU07070.1	ESGALSVAKLQYLVLDEADLVLSYGYDEDMENIARSLPKGGVQTTMMSATLVSDELDTLK
FG02752.1 	NSSALSIDKLTHLILDEADLVLSYGYSEDLENLSRSVPKG-VQVMMMSATLS-DEVDTLK
AN6374.1  	GTSALSLENLTHLVIDEADLVLSYGYEDDINALSKAIPRG-VQTFLMSATLT-AEVDTLK
          	 :.***: :* :*::***********.:*:: :::.:*:* **. : ****   *:****

MG04993.1 	GIFLRDPVLLDLEEPD-AEGSEITQYIVK2CGEDEKFLLAYILFKLQLIKGKVIIFVADV
NCU07070.1	GFFCRNPTMLDLKEEFSNEDEKLTQFYVK~CGEDDKWLISYLIFKLQLIKGPCLVFVADI
FG02752.1 	GIFRRDPTLLDLKEKE-AEGEGITQFVAK2CGEDEKFLLAYVIFKLKLIKGKCIIFVSDI
AN6374.1  	GLFCRSPVILKLEDKD-DHGAGVSQFVVK2CAEDEKFLLTYVIFKLQLIKGKVIIFVGDI
          	*:* *.*.:*.*::    ..  ::*: .* *.**:*:*::*::***:****  ::**.*:

MG04993.1 	DRCYRLKLFFEQFGIRSCILNSELPVNSRISVVEEFNRNVYDIIIASDE-NEMMGDEDRP
NCU07070.1	DRAYRLKLFFEQFSIRSCVLNSELPINTRIKIIEEFNRGIYDIIIASDERSEVFLEDEKT
FG02752.1 	DRCYRLKLFFEQFGIRSCILNSELPLNSRVHVVEEFNRHVYDIIIAADEKNEMLGDDEEP
AN6374.1  	DRSYRLKLFLEQFGIKSCILNSELPVNSRIHVVEEFNKGVYDIIIAADE-QEVLGVSKSR
          	**.******:***.*:**:******:*:*: ::****: :******:** .*::  ..  

MG04993.1 	APNGDGKEEAEVDKKHENEDEGQDGEASKAAPRPKKKRKMDRKRDKQYGVSRG~IDFKNV
NCU07070.1	EE----KKEEQGEKK---EGD--------EKKNGKGKKKKGRR-DQEYGVSRG1IDFKNV
FG02752.1 	AE----TAEAQDDAKKSNEGDD----AETEAKRPKKKAKKSKGGDKEYGVSRG1VDFKKV
AN6374.1  	KS----KDATEGDDELLSDEDEE--TSAKAASTRTDKRRKLSSKEKDYGISRG~IDFQNV
          	      .   : : :  .: : .   : .     . * :     :::**:*** :**::*

MG04993.1 	AVVVNFDLPLSSTSYTHRIGRTGRAGQAGMVLSFYVPKELFRKHIPTSIDSAENDEKVLA
NCU07070.1	AAVINFDMPTSSSSYIHRIGRTARAGRAGIALSMVVPHDLFGKHKPTSIKQCEKDEKVLA
FG02752.1 	SAVINFDLPTTASAYTHRIGRTARAGQTGMALSFVVPKDLYRKHMPTSTPACENDEKIMA
AN6374.1  	ACVLNFDLPTSSKSYTHRIGRTGRGGKTGMALSFVIPADKYGKHKPTSISSAKHDEAVLA
          	: *:***:* ::.:* ******.*.*::*:.**: :* : : ** ***   .::** ::*

MG04993.1 	RVIKQQKKLGREIKPYNFDRDQVESFRYRMNDA~LRAVTKIAVREARTRELRQELLKSEK
NCU07070.1	KVMRQQAKLNRKLEPYNFNKDQMEAFRYRMNDA1LRAVTKVAIREARTRELRQELLRSET
FG02752.1 	RIIRQQAKRDKEVKPYNFNMKQVDPFRYRMNDA~LRAVTKVAIREARTRELRQELLKSEK
AN6374.1  	KIIKRQAKLGHEVKPYHFDMTQVDAFRYRMSDA~LRAITRLAIQEARAREIRQELVKSEK
          	:::::* * .::::**:*:  *::.*****.** ***:*::*::***:**:****::**.

MG04993.1 	LKR~HFEENPAELQHLVRHDGELRT-ARANPELRHVPDYLLPKEGRKGLSAAEIGFVPLR
NCU07070.1	LKR~YFEENPHELSHLR-HDGELGTKMRQQAHLKHVPDYLLPQDGKNALTETQIGFVPFK
FG02752.1 	LKR2YFEENPTELSHLR-HDGELRT-ARQQAHLKHIPEYLMPKDGKQALTED-VGFVAMR
AN6374.1  	LKR~HFEENPEELRQLR-HDGELRA-ARIQPHLKHIPEYLMPAKGKKGISNEDVGFVSLR
          	*** :***** ** :*  ***** :  * :..*:*:*:**:* .*::.::  .:***.::

MG04993.1 	K----SSDRQKKGRFFKKGGARSFKAGGR-KPDPLKTFKAKRKTK
NCU07070.1	KQGDDKKTRGKKGAKGGKGGHGKYKKGPGGRKNVLKTFRVRK---
FG02752.1 	K---DKKGKGKK----GRG----FKVGSR-KRDPLKTFKARRKTK
AN6374.1  	KTGPENRIRKARDRNRGRG-----KKPGR-KIDPLKTFNRGRK--
          	* . ..  :  :.    :*     *     : : ****.  :.
```
